# Development and Evaluation of an Immersive Virtual Reality Application for Road Crossing Training in Older Adults

**DOI:** 10.3390/geriatrics10040099

**Published:** 2025-07-24

**Authors:** Alina Napetschnig, Wolfgang Deiters, Klara Brixius, Michael Bertram, Christoph Vogel

**Affiliations:** 1 Department of Health Sciences, Bochum University of Applied Sciences, Gesundheitscampus 6–8, 44801 Bochum, Germany; wolfgang.deiters@hs-bochum.de; 2 Institute of Circulatory Research and Sports Medicine, German Sport University Cologne, Am Sportpark Müngersdorf 6, 50933 Cologne, Germany; brixius@dshs-koeln.de; 3 vobe GbR, Kreuzweg 19, 49134 Wallenhorst, Germany; michael@vobe.digital (M.B.); christoph@vobe.digital (C.V.); 4 Institute of Mixed Reality and Visualization (MiReVI), Hochschule Düsseldorf—University of Applied Sciences, Münsterstraße 156, 40476 Düsseldorf, Germany

**Keywords:** immersive technologies, senior citizens, application development, training program, crossing the road, health in old age

## Abstract

**Background/Objectives:** Aging is often accompanied by physical and cognitive decline, affecting older adults’ mobility. Virtual reality (VR) offers innovative opportunities to safely practice everyday tasks, such as street crossing. This study was designed as a feasibility and pilot study to explore acceptance, usability, and preliminary effects of a VR-based road-crossing intervention for older adults. It investigates the use of virtual reality (VR) as an innovative training tool to support senior citizens in safely navigating everyday challenges such as crossing roads. By providing an immersive environment with realistic traffic scenarios, VR enables participants to practice in a safe and controlled setting, minimizing the risks associated with real-world road traffic. **Methods:** A VR training application called “Wegfest” was developed to facilitate targeted road-crossing practice. The application simulates various scenarios commonly encountered by older adults, such as crossing busy streets or waiting at traffic lights. The study applied a single-group pre-post design. Outcomes included the Timed Up and Go test (TUG), Falls Efficacy Scale-International (FES-I), and Montreal Cognitive Assessment (MoCA). **Results:** The development process of “Wegfest” demonstrates how a highly realistic street environment can be created for VR-based road-crossing training. Significant improvements were found in the Timed Up and Go test (*p* = 0.002, d = 0.784) and fall-related self-efficacy (FES-I, *p* = 0.005). No change was observed in cognitive function (MoCA, *p* = 0.56). Participants reported increased subjective safety (*p* < 0.001). **Discussion:** The development of the VR training application “Wegfest” highlights the feasibility of creating realistic virtual environments for skill development. By leveraging immersive technology, both physical and cognitive skills required for road-crossing can be effectively trained. The findings suggest that “Wegfest” has the potential to enhance the mobility and safety of older adults in road traffic through immersive experiences and targeted training interventions. **Conclusions:** As an innovative training tool, the VR application not only provides an engaging and enjoyable learning environment but also fosters self-confidence and independence among older adults in traffic settings. Regular training within the virtual world enables senior citizens to continuously refine their skills, ultimately improving their quality of life.

## 1. Introduction

Innovative technologies, such as augmented reality applications, are undergoing remarkable development and integration into various aspects of daily life [1,2]. These technologies—including virtual reality (VR), augmented reality (AR), and mixed reality (MR)—offer novel ways of interacting with digital content and enhancing user experiences across a wide range of applications [3]. Augmented reality (AR) overlays digital information onto the real world, while virtual reality (VR) creates fully virtual environments. Mixed reality (MR) combines both approaches, allowing real and virtual objects to interact in real time. These technologies fundamentally change perception and information processing and are increasingly used by people of all ages [2]. Their applications range from games to cognitive and entertainment tools.

One prominent subset of these technologies is serious games—computer-based programs designed to impart knowledge and skills engagingly and entertainingly [1]. Numerous studies have demonstrated the effectiveness of serious games in boosting user motivation and engagement while fostering significant improvements in acquired skills. For instance, Gentry et al. (2019) [1] found that immersive VR interventions can alleviate pain and enhance the quality of life for older adults. Additionally, gamification has emerged as a complementary strategy to increase engagement in non-game contexts. By incorporating game-like elements such as points, leaderboards, and rewards into applications, gamification motivates users to adopt specific behaviors or achieve learning goals [4]. While gamification enhances existing activities with playful incentives, serious games focus on standalone games with clearly defined educational objectives [5]. Together, these developments highlight the transformative potential of serious games and gamification as innovative tools for knowledge transfer and skill development, fostering active engagement with complex topics. In the realm of virtual reality (VR), serious games are increasingly being employed to deliver content in both knowledge-based and immersive ways [6]. In gerontology, these technologies are being utilized to support older adults in various areas of life. Virtual applications have been applied in memory work, movement therapy, pain management, relaxation techniques, and the promotion of everyday activities known as Activities of Daily Living (ADLs) [7]. Training in virtual environments offers senior citizens opportunities to maintain or even improve their abilities. Research has shown significant positive effects of serious games on the cognitive and motor skills of older adults. Table A1 (see Appendix A, [8,9,10,11,12,13]) provides a comparative overview of current VR-based training applications for older adults. The studies analyzed show a variety of approaches, e.g., with regard to movement (e.g., sitting, walking, cycling) and interaction (from simple controllers to motion tracking). For example, Kustanovich et al. (2023) [5] demonstrated that serious games significantly improved memory and cognitive functions in seniors with dementia over a ten-week period. Through targeted exercises within a playful environment, participants not only trained their cognitive abilities but also preserved their everyday skills. Similarly, Anderson-Hanley et al. (2012) [6] provided evidence that serious games used in motor rehabilitation positively impact balance and coordination among older adults. Movement-based games—commonly referred to as exergames—not only promote physical fitness but also enhance user engagement and motivation.

Moreover, an exploratory pilot study conducted as part of the “SenseCity” project by Joanneum Research Digital in Austria examined the impact of VR applications on the quality of life among older adults [8]. Participants reported positive emotions and memories evoked by virtual experiences such as visiting a park or taking a gondola ride through Venice. These virtual experiences not only foster social interaction but also contribute to emotional stability. Randomized controlled trials, some with large samples, show positive effects on balance, cognition, emotional well-being and fall prevention. Adaptive and progressively structured VR systems that specifically promote both physical and cognitive functions appear to be particularly effective. The use of serious games and VR technologies thus presents promising approaches for supporting older adults in their daily lives. These technologies can help enhance cognitive and motor skills while significantly improving overall well-being and quality of life for senior citizens. The VR training directly targets age-related cognitive, visual, and motor changes by incorporating tasks that require attention and decision-making, simulating diverse visual conditions to support perception, and engaging users in physical movement and gestures to enhance balance and coordination. Given demographic changes and the growing proportion of older adults worldwide, it is essential to develop innovative solutions tailored to the specific needs of this population group. In summary, VR technologies and serious games represent effective tools for promoting independence among older adults while helping them maintain or improve their daily living skills. Future research should prioritize further development of these technologies while evaluating their potential applications across diverse contexts.

Building on these findings, this study focuses specifically on the development and evaluation of the VR training application “Wegfest”, which addresses the everyday challenge of road crossing among older adults. The findings outlined above emphasize the importance of virtual reality (VR) in training everyday activities. One such activity, crossing a road, serves as the central use case for this study. This use case is grounded in concerning statistics: in Germany, the incidence of road traffic accidents among senior citizens increases with age. According to statistical data, 12% of those injured and 29% of those killed in traffic accidents were aged 65 or older [14]. The consequences of such accidents are often more severe for older adults compared to younger road users due to their reduced physical resilience [15]. Crossing a road safely presents a significant challenge for many older adults. From the age of 75 onwards, the risk of accidents during road crossing increases substantially, with incorrect behavior frequently identified as the primary cause [16,17]. Age-related changes in motor skills, cognitive function, and visual acuity exacerbate these difficulties. Motor impairments often manifest as reduced walking speed and difficulties adapting to varying situations [18,19,20]. Additional motor changes include shorter stride lengths and compensatory movements stemming from balance disorders [21]. Furthermore, postural control becomes increasingly vulnerable to disruptions with age, while gait and posture planning are impaired [18,19]. These motor deficits are particularly problematic when navigating roads with high vehicle speeds [22].

The main objective of this study was to investigate the feasibility, acceptance, and initial effectiveness of a newly developed virtual reality training program (“Wegfest”) designed to promote safe road crossing among older adults. The focus was on whether the VR training could positively influence the participants’ functional mobility, fear of falling, and subjective sense of safety. In addition, initial indications of the transferability of the VR training content to everyday skills were to be obtained. We hypothesize that immersive VR training with adaptive road-crossing scenarios will improve functional mobility, reduce the fear of falling, and increase subjective safety among older adults.

## 2. Materials and Methods

### 2.1. Development of VR Application “Wegfest”

**Figure 1:**
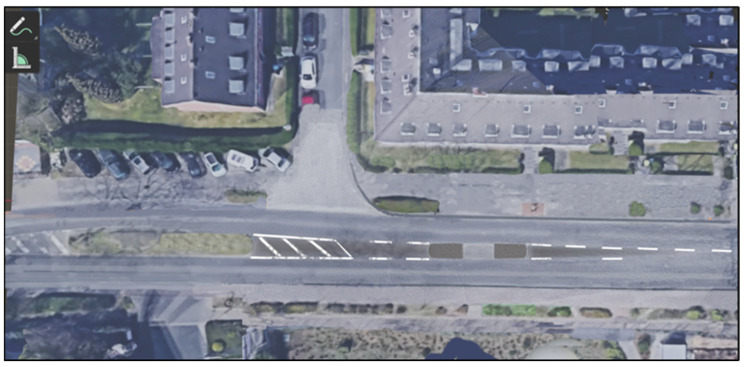
Google Maps screenshot, crossing situation traffic island in Düsseldorf (Google, 2009).

**Figure 2:**
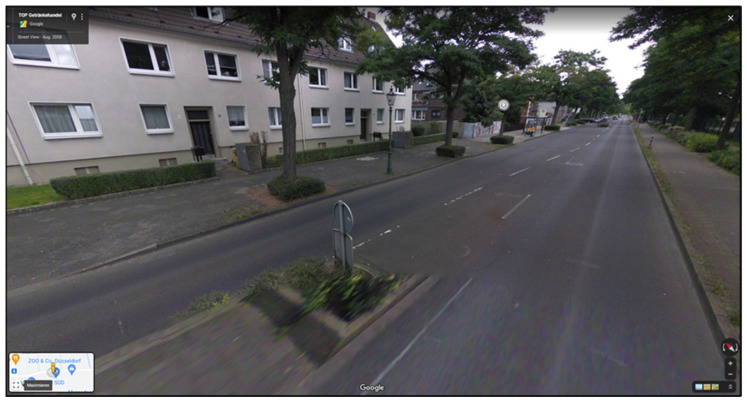
Screenshot of Google Street View Maps, crossing situation traffic island in Düsseldorf on Person View (Google, 2009).

**Figure 3:**
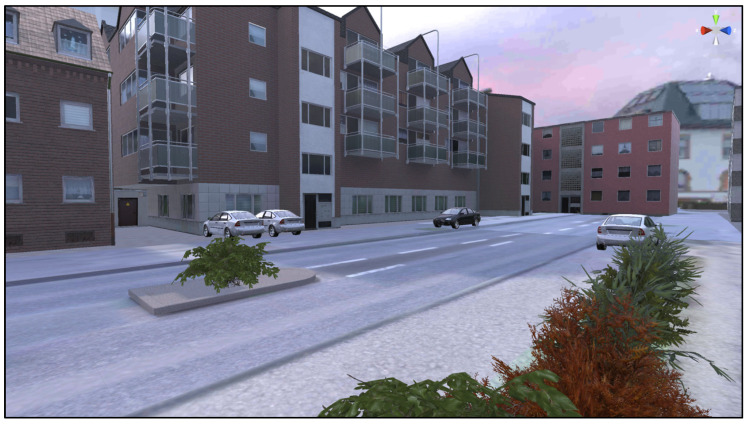
Screenshot of scene Wegfest, crossing situation traffic island based on Google Street View Maps (own illustration).

To address these challenges, a VR training application called “Wegfest” was developed to teach safe road-crossing techniques. This application uses various virtual road scenarios to help senior citizens learn how to cross streets while considering multiple factors. “Wegfest” qualifies as a serious game because it integrates explicit learning objectives—namely, safer road-crossing behavior—into an interactive virtual environment, guided by principles of motivation and skill acquisition. The development process utilized the Unity Engine and was carried out by the vobe GbR team using C# programming language. The modeling phase involved designing individual scenes featuring diverse environments and objects. To create a realistic virtual environment, street scenarios from German cities—including Cologne, Bochum, Münster, and Düsseldorf—were selected as templates for development. These street situations were captured using Google Maps and subsequently implemented into Unity (see Figure 1, Figure 2 and Figure 3). The use of Google Maps imagery for scene design was limited to publicly available references for orientation. Nevertheless, future iterations of the application should address potential copyright and licensing considerations when reproducing or distributing map-based environments. The virtual environments aim to replicate real-world conditions as closely as possible to ensure effective training outcomes. The VR environment was constructed at a 1:1 scale, with each Unity unit equating to one real-world meter. This enabled accurate replication of real-world spatial dimensions, such as the 6.2 m curb-to-curb distance measured at a selected Düsseldorf crossing. Unity’s built-in measurement tools and custom scripts further ensured precise distance calculation and collision detection, guaranteeing faithful simulation of real-world conditions.

The development of the VR application was based on a multi-stage participatory process that integrated various user-centered design approaches, incorporating expert insights as well as feedback from the target group of older adults through a series of workshops (cf. [22]). Feedback from participants during the tutorial phase was systematically collected and directly informed several aspects of the application’s design, including the simplification of instructions, optimization of gesture recognition, enhancement of navigation aids, and improvements to comfort and usability features. During workshops, feedback was collected from seniors regarding requirements for VR and the “Wegfest” application (cf. [7,22]).

**Figure 4:**
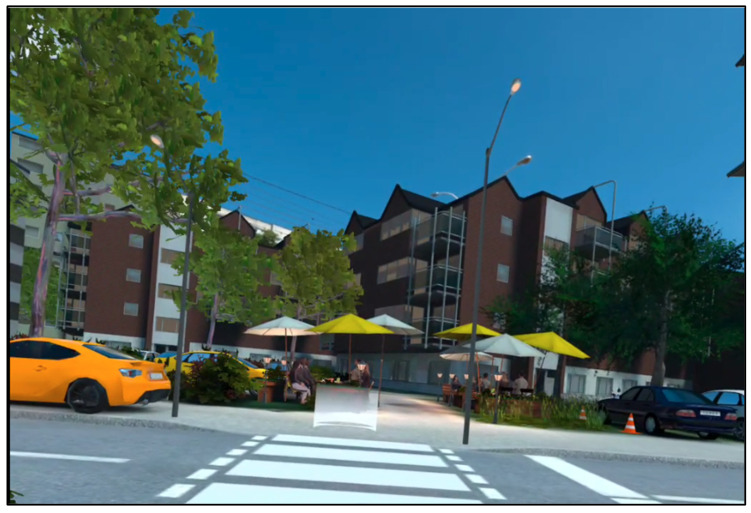
Daytime scene at the “Wegfest” (own illustration). Elements shown include sidewalks, traffic lanes, animated pedestrians, and surrounding urban environment.

The scene design incorporates paid assets, including animated characters capable of performing interactions such as walking and gesturing. Figure 4 presents a screenshot of a daytime scene from “Wegfest,” highlighting the integration of these dynamic elements into the virtual environment. These assets contribute to creating a realistic and immersive experience for users.

The Meta Quest 2 head-mounted display (HMD) was utilized for this project. Interactions in virtual reality (VR) were conducted by physically walking and gesturing, supported by hand-tracking technology. Continuous pre-tests, carried out by programmers and senior staff, facilitated iterative adjustments during the application development process. Beginning in July 2022, the existing version of “Wegfest” was tested with a sample of *n* = 8 older adults. Participants were recruited through personal networks and the Bischoff Physiotherapy Practice in Düsseldorf. After each pre-test phase, feedback from participants was collected and integrated into the application design. Following the fourth pre-test with older adults, the application was deemed acceptable by users, enabling its prototype to be used for the subsequent intervention study.

Before starting their first training session, users completed an integrated tutorial within “Wegfest.” This tutorial was designed to familiarize them with interaction patterns and navigation in the virtual environment. It allowed participants to individually explore various movement sequences and helped them acclimate to VR, a new medium for many senior citizens. The tutorial explained the functions of the application, outlined required interactions, and assessed their applicability. In addition to serving as a tool for onboarding participants, it also evaluated their experience of wearing the VR headset while physically walking. Once participants successfully completed the tutorial, the first scene of a predefined iteration was loaded. An iteration consisted of multiple consecutive street-crossing scenarios defined via a web application. The web application comprised a front end, back end, and database that organized structured information electronically. An Internet connection was required for data transfer. The modular structure of various scenes enabled diverse applications for road-crossing training in VR environments. The scenes incorporated various vehicle models, including motorized and non-motorized vehicles.

A proximity-based collision prevention system utilizes two spatial zones:

- Region of Warning (ROW): Activates alerts when vehicles approach predefined distance thresholds.
- Region of Interest (ROI): Triggers scene freezing during bounding box overlaps between users and vehicles to simulate near-misses/collisions.

Vehicles employ dynamically adjustable speed profiles (30–70 km/h for motorized vehicles, 10–20 km/h for non-motorized vehicles) with realistic acceleration/deceleration patterns. Bounding box detection ensures real-time spatial monitoring, while the dual-zone model balances hazard prevention with traffic flow realism.

**Figure 5:**
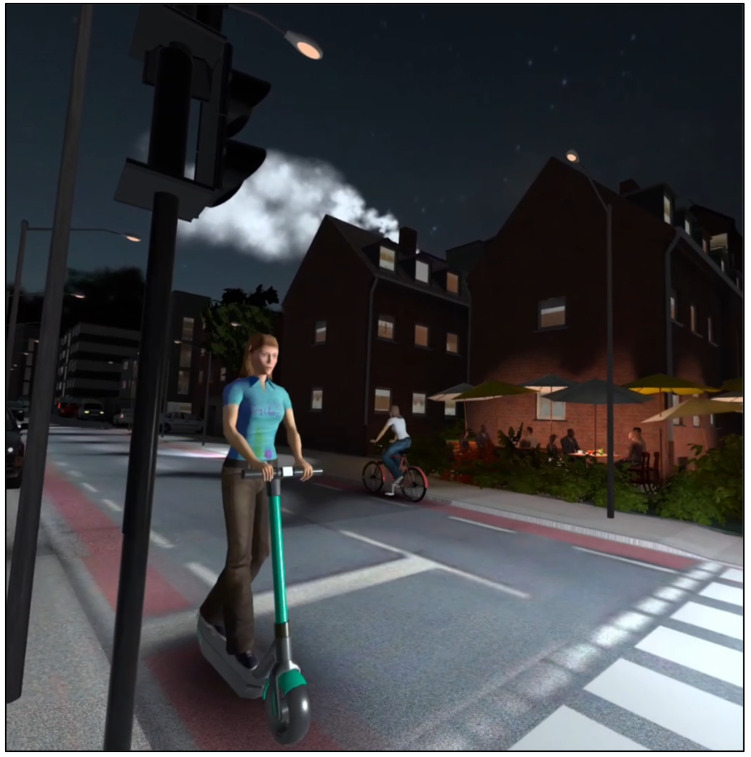
Screenshot of a road fixed with non-motorized vehicles (own illustration). Elements shown include bicycle lanes, traffic lights, pedestrian crossings, a café, and ambient sounds, including those from electric vehicles.

Modeling based on specific distance calculations—such as Region of Warning (ROW) and Region of Interest (ROI)—was employed to prevent virtual collisions and ensure user safety. A white circle on the opposite side of the curb acted as a target marker for participants. Scene parameters were configured to reflect real-world conditions during road crossings and included acoustic and visual stimuli. Acoustic inputs featured vehicle sounds (e.g., electric or motorized vehicles) as well as ambient noises such as voices or music. Visual inputs included varying crossing distances (e.g., lane width), crossing aids (e.g., traffic lights, crosswalks, traffic islands), and different times of day (e.g., daytime, twilight, night). Figure 5 displays a screenshot from “Wegfest,” showcasing an e-scooter or bicycle within a nighttime scene.

The selection of scenes for each iteration is guided by specific parameters. To ensure adaptive progression in training, the requirements and level of difficulty in individual training units are systematically increased. The configuration of scenes within each iteration is determined based on these parameters. Comprehensive research is conducted to evaluate the relevance of individual parameters as influencing factors in road-crossing scenarios. This evaluation enables parameter weighting and serves as the foundation for variability in training design.

The VR scenarios were explicitly designed to simulate key factors influencing real-world road crossing, including traffic density and vehicle types, crossing aids, environmental and auditory conditions, crossing distances, realistic vehicle behavior, adaptive scenario difficulty, and the need for physical and gesture-based user interaction.

Gesture-based interaction was selected to minimize the need for handheld controllers, simplify user input, and increase accessibility for older participants who may be unfamiliar with complex devices. Gesture control was used in the presented study for three main purposes: first, it served to activate a virtual button that appeared whenever a “near miss” or accident occurred, allowing the scene to be replayed. Second, raising the hands simulated a real-world street crossing, although in the current prototype this action only contributed to increasing immersion and did not carry any functional link. Additionally, after each scene, users could select one of up to five answer options to questions (e.g., about perceived safety) using hand gestures.

To capture user interactions, the position and orientation of the hand as well as selected gestures (such as pressing a button) were recorded using a hand-tracking SDK from Meta Quest 2. This system is based on computer vision for estimating hand position. In preliminary tests, the system achieved a recognition accuracy of about 90% under standard lighting conditions. Errors occurred primarily when the hands were outside the field of view or moved too quickly. To minimize this, users were instructed to perform slow, deliberate gestures, and a visual feedback signal was provided upon successful gesture recognition.

The training framework is structured around a value system in which scenes are configured by selecting parameters with corresponding difficulty gradations (e.g., 1 = easy; 3 or 4 = difficult) (see Table 1). Progression to a scene with a higher challenge level occurs only after the user successfully completes a road-crossing attempt that is both accident-free and safe.

Table 1 provides an overview of the various parameters used to configure scenes in the VR application “Wegfest.” By summing the values assigned to each parameter, a total score is calculated, which represents the difficulty level of the respective scene. Table 2 presents an example of a training session consisting of seven distinct scenes with varying levels of difficulty; the cumulative sum of the parameters for this session is 48.

A scene was considered completed once the participant’s avatar reached the designated target marker on the opposite curb without any collision incident. The application recorded the duration required to complete each scene per session. While additional movement metrics were not captured in this study, future research may incorporate more detailed data collection and advanced technologies. The current project did not focus on tracking individual position data.

### 2.2. Research Design

This study was designed as a single-arm, prospective intervention study (pre-post design) to investigate the feasibility and initial effectiveness of the VR application “Wegfest” for training older adults to cross the street safely.

The focus was on analyzing functional and subjective changes after an eight-week VR-based training program. Objective clinical parameters—such as mobility (TUG), cognitive performance (MoCA), and fall-related self-efficacy (FES-I)—as well as subjective assessments of perceived safety when crossing roads were collected.

To examine the effects of the VR application “Wegfest” on the physical and psychological parameters of senior citizens, an intervention study was conducted. The primary aim was to evaluate the impact of VR training designed to simulate various scenarios for crossing roads safely. Due to the exploratory nature of the study, a control group was deliberately not included. The results will serve as a basis for further controlled studies and will test the transferability of the VR-based training environment to practical application contexts.

### 2.3. Participants

The sample size was determined using a G*Power (Version 3.1.9.7) analysis, assuming a large effect size (*dz* = 0.8), a significance level of *α* = 0.05, and a test power of 1 − *β* = 0.95. Of the *n* = 29 recruited participants, *n* = 20 were included in the final analysis. Three participants withdrew due to scheduling conflicts or personal reasons, four attended training sessions too infrequently to meet protocol requirements, and two discontinued after experiencing mild cybersickness. Only participants who completed the full intervention and all assessments were analyzed, ensuring data integrity and comparability. This complete-case approach is consistent with best practices in feasibility and pilot studies.

Participants were selected based on specific inclusion and exclusion criteria. Inclusion criteria required individuals to be aged 70 years or older, without a fall risk, living independently, and physically fit. Exclusion criteria included contraindications for VR use (e.g., epilepsy, vertigo), severely limited walking ability, significant balance issues, cognitive impairments, bedridden status, severe cardiovascular diseases, use of mobility aids (e.g., walker or cane), severe visual impairments or eye diseases, and significant hearing loss. These criteria ensured participant safety and study validity. The participants ranged in age from 71 to 81 years (mean age: 74.95 ± 3.17 years). Of the participants, *n* = 12 were male and *n* = 8 female; all were individually assessed for health suitability before participation. All participants provided informed consent, and the study received approval from the ethics committee of the German Sport University Cologne (reference nb. 095/2022).

### 2.4. Assessments

Prior to the VR intervention, several assessments were conducted to ensure the physical and mental suitability of participants:

- Timed Up and Go (TUG) Test: This test evaluated mobility and fall risk by assessing functional performance in older adults, focusing on flexibility and balance [23]. The TUG test is a widely used clinical assessment that measures a person’s functional mobility. Specifically, it evaluates the time (in seconds) it takes for an individual to stand up from a seated position, walk a distance of three meters, turn around, walk back to the chair, and sit down again. The TUG test provides an objective measure of balance, gait speed, and lower limb function, and is commonly used to assess fall risk in older adults. A cut-off value of <20 s indicated unrestricted mobility in daily activities. The TUG demonstrated excellent reliability (interrater reliability: ICC = 0.99; intrarater reliability) and strong validity correlations with established measures such as the Berg Balance Scale (*r* = −0.81), Barthel Index of ADL (*r* = −0.78), and gait speed (*r* = −0.61) [24].
- Falls Efficacy Scale-International Version (FES-I): The FES-I assessed fall-related self-efficacy using a 16-item questionnaire that evaluated complex functional activities and social aspects of self-efficacy [25]. This instrument demonstrated high internal consistency (*Cronbach’s α* = 0.96) and excellent test-retest reliability (*r* = 0.96).
- Montreal Cognitive Assessment (MoCA): The MoCA screened for cognitive deficits across domains such as memory, executive functions, attention, and visuospatial skills. It was particularly relevant for evaluating cognitive abilities required for VR use in “Wegfest” scenarios [26]. A cut-off score > 26 points indicated normal cognitive function. The MoCA showed strong psychometric properties with reliability (*r* = 0.89) and sensitivity (80%) at this threshold [27].

Additional questionnaires assessed VR suitability and participants’ subjective perceptions of safety when crossing roads in virtual environments. A visual analogue scale (VAS, range 0–10) was used to assess participants’ subjective sense of safety when crossing roads in virtual environments.

### 2.5. Intervention Process

The intervention took place between October and December 2022, with participant recruitment beginning on 15 August 2022. Recruitment involved distributing information sheets at Bischoff Physio-therapy Practice, RKM 740 Physiotherapy Facility in Düsseldorf, senior centers in Bochum (in collaboration with NRW Police), and personal networks in Düsseldorf and Cologne.

For “Wegfest,” a rectangular physical play area of at least 8 × 4 m was defined. This size was chosen to accommodate the typical space requirements of a street intersection with two lanes and sidewalks, and it provides an additional lateral safety margin of 2 m for evasive movements. Crossing the street is simulated by physically walking back and forth along an imaginary line between the start and target markings; upon reaching the target, a 180° turn is performed and the next scenario is started. The intervention was conducted in a gymnasium or large community hall to ensure sufficient space for movement. The presence of a person trained in fall prevention allowed the virtual safety boundary to be disabled, thereby enabling a larger movement area. The intervention duration and structure were informed by comparable studies (e.g., Mirelman et al. (2016) [10]; Anderson-Hanley et al. (2012) [6] to ensure clinical feasibility and user engagement. The training design of “Wegfest” followed the recommendations of the American College of Sports Medicine [28], which suggest incorporating a maximum of 8 to 10 exercises per session. In line with this and considering the innovative VR-based approach, each session included up to seven scenarios, with each scenario conceptualized as an individual exercise. A buffer was integrated into the program to allow for repetitions, enabling participants to safely re-encounter potentially hazardous situations or accidents. Training sessions lasted approximately 20 to 30 min.

During these sessions, participants practiced crossing roads under various conditions in VR scenarios that gradually increased in complexity. Participants were individually supervised throughout the sessions to monitor their reactions closely and allow for immediate intervention if necessary. All intervention sessions included an in-app tutorial for acclimatization and were conducted under the supervision of a trained study team member. This ensured participant safety, supported adaptation to VR, and allowed for immediate assistance in case of discomfort or technical issues.

The study received ethical approval from the German Sport University Cologne. Data analysis was performed using IBM SPSS Statistics and Microsoft Excel. Statistical analyses were performed using IBM SPSS Statistics (Version 28), a standard tool for handling small sample, exploratory studies with parametric and non-parametric approaches. This software was used for all descriptive and inferential analyses, including normality testing, calculation of effect sizes, and corrections for multiple comparisons.

Ethical standards were upheld through written informed consent and data protection agreements.

## 3. Statistical Analysis and Results

A total of 20 participants (mean age: 72.4 ± 5.8 years; 65% female) completed the VR intervention and all assessments. The drop-out rate was 31.03%. Reasons for exclusion included a TUG value exceeding the cut-off threshold (>23 s), motion sickness experienced by two participants who discontinued training prematurely, and irregular attendance (<75% of sessions) by six participants.

The effects of the VR intervention on clinical endpoints are summarized in Table 3.

Normality of distribution for all outcome variables was assessed using the Shapiro–Wilk test. Based on these results, parametric or non-parametric tests were applied accordingly. The evaluation of the Timed Up and Go (TUG) test data indicated a normal distribution for both measurement time points; therefore, a paired *t*-test and the Wilcoxon signed-rank test were used for analysis. According to Cohen’s guidelines, values of *d* = 0.2, 0.5, and 0.8 correspond to small, medium, and large effects, respectively. In this study, the *t*-test revealed a significant difference between the measurement time points (*p* = 0.002), with a *Cohen’s d* of 0.784—indicating a large effect size approaching the conventional threshold of 0.8.

The mean TUG time improved significantly from 12.1 s (*SD* = 2.2) before the intervention to 10.6 s (*SD* = 2.0) after the intervention (*p* = 0.002).

The Montreal Cognitive Assessment (MoCA) results showed no significant difference (*p* = 0.56), suggesting no change in the subjects’ cognitive abilities.

A significant result was obtained for the Falls Efficacy Scale-International Version (FES-I) (*p* = 0.005, *z* = −2.818), indicating an improvement in self-efficacy regarding fear of falling. These results support the effectiveness of the VR intervention in terms of functional performance and fall prevention. For non-parametric analyses, effect sizes were calculated using *r* = z/√N to allow the interpretation of practical relevance. To account for multiple comparisons, the Bonferroni correction was applied, adjusting the significance threshold to *α* = 0.05/3 = 0.0167.

The subjective assessment of the participants was evaluated using the Wilcoxon signed-rank test at the ordinal scale level. The analysis showed a significant change between the pre-assessment and the post-assessment (*p* < 0.001, *z* = 7.157). The participants reported a significant improvement in their subjective perception of their own safety when crossing the road after completing the VR intervention.

### Summary of the Results

The results of the study indicate significant positive effects of the VR intervention, both in objective performance parameters (TUG and FES-I) and in the subjective perception of the participants. The improvement in the subjects’ self-efficacy and subjective safety supports the assumption that VR-based training programs can positively influence the functional performance and sense of safety of senior citizens.

## 4. Discussion

The VR application “Wegfest” has selected road crossing as a central use case because this everyday activity is of great importance for senior citizens. Statistics show that older adults are frequently involved in accidents due to improper road crossing [17]. The causes of this increased risk are multifaceted and include age-related changes in cognition, vision, and motor skills [18,29]. This issue is also addressed in the International Classification of Functioning, Disability and Health (ICF), which covers various aspects of walking and locomotion [30]. Although there has been a slight decline in traffic accidents among senior citizens in recent years, this is attributable to several factors. For example, isolation and reduced mobility during the COVID-19 pandemic led to fewer accidents [31]. Increased road safety measures, such as more frequent speed cameras and speed limits, have also contributed to a reduction in the number of accidents [32,33]. Nevertheless, road safety for senior citizens remains a key concern, especially given demographic changes and the aging population [34].

In contrast to conventional road safety education programs—which often rely on group-based instructions or static demonstrations—VR offers interactive, adaptive, and experiential learning with higher ecological validity. Compared to existing VR training applications for older adults, “Wegfest” introduces several novel features. While earlier systems often relied on stationary exercises (e.g., seated setups or limited interaction) or predefined, non-adaptive scenarios (see Table A1, Appendix A), “Wegfest” incorporates a highly realistic, action-oriented road-crossing environment that requires full physical movement such as walking and hand gestures, coupled with scenario-specific progression. The application was designed based on actual urban traffic settings and integrates hand-tracked gesture interaction, as well as complex traffic dynamics including motorized and non-motorized vehicles, acoustic stimuli, and varied lighting conditions. Notably, the adaptive configuration of scenes using parameterized difficulty levels (e.g., traffic volume, crossing aids, time of day) allows for personalized training progression—an approach that has been largely absent from prior studies. In addition, the combination of immersive physical engagement, interactive feedback, and realistic hazard prevention mechanisms (e.g., collision warning and freezing via bounding-box logic) enables context-rich simulation of complex everyday challenges. This may yield a higher potential for real-world transfer compared to previous systems.

This approach aligns with recent research emphasizing the importance of ecological validity and adaptive complexity in VR-based mobility training [35]. For example, Ali et al. (2023) [36] argue that immersive and ecologically valid environments enhance both cognitive and motor engagement in older adults. Similarly, González-Erena et al. (2025) [3] highlight the value of multimodal VR systems that dynamically adapt to the user’s performance and environmental variables in promoting transferability of training effects to real-life mobility situations.

The development of the VR application “Wegfest” raises several important points for discussion. First, there is the question of the effectiveness of such VR training programs compared to traditional training methods. Studies have shown that VR training can have a positive impact on the cognitive and motor skills of older adults. For example, Benham et al. (2019) [37] reported that immersive VR interventions not only improve physical well-being but can also boost participants’ self-confidence. Another aspect is the adaptability of the VR application to individual needs. The ability to simulate different scenarios—such as crossing a road in different lighting or traffic conditions—could enable seniors to train specific challenges and improve their skills in a targeted manner [7]. This could not only increase road safety but could also promote general self-confidence in dealing with everyday situations. Additionally, it is important to consider how realistic the VR representations are and how they influence the user’s perception. Benoit et al. (2015) [38] emphasized the influence of VR design on users’ sense of presence. A high degree of realism could help senior citizens to better prepare for real-life situations. However, there is also a risk that excessive familiarity with the virtual environment could lead to an overestimation of their abilities in real life. Another point of discussion concerns the accessibility and acceptance of VR technologies among older adults. Many seniors may be unfamiliar with modern technology or have difficulty operating VR devices [39]. Therefore, it is crucial to develop training programs that facilitate the use of VR applications while addressing concerns about possible side effects such as kinetosis [40]. To improve accessibility and reduce side effects such as cybersickness, future developments should include customizable comfort settings, gradual onboarding, shorter sessions with breaks, both seated and standing modes, enhanced visual stability, and clear visual and audio cues. These measures can help ensure safe and enjoyable participation for a wide range of older adults. Thus, it can be said that the use of virtual reality to improve road safety for seniors offers promising approaches. However, further research and discussions are needed on design criteria and on the integration of such technologies into existing programs to support older adults. The results of these discussions could help to create guidelines for the development of effective and user-friendly VR applications.

This study was conducted as a feasibility and pilot study to evaluate the usability, acceptance, and initial effectiveness of a VR-based intervention in older adults. Given the exploratory nature of the study, a single-group design was chosen, focusing on within-subject comparisons rather than between-group analyses. While this approach limits our ability to draw causal conclusions regarding the intervention’s effectiveness, it provides valuable insights into the intervention’s feasibility and identifies areas for improvement in future, more controlled research.

The Timed Up and Go (TUG) test was used to assess changes in functional mobility following the VR intervention. The mean TUG time improved from 12.1 s (*SD* 2.2) pre-intervention to 10.6 s (*SD* 2.0) post-intervention, corresponding to a mean reduction of 1.5 s. According to the established literature, a change of 1.0–2.0 s in TUG time is considered the minimal clinically important difference (MCID) for older adults at risk of falls [41,42]. The improvement observed in our study thus meets the lower threshold for clinical significance, suggesting that the intervention may have a meaningful impact on participants’ functional mobility.

This presentation on the development of VR applications in connection with safe road crossing emphasizes the growing relevance of virtual reality in the area of “healthy aging.” Serious games, in particular, offer innovative approaches to support older adults in their daily mobility. The integration of playful elements into serious learning content not only promotes user engagement but also improves learning success through immersive experiences. The relevance of these technologies is underpinned by empirical studies. The results suggest that VR interventions can not only provide therapeutic benefits—such as reducing pain sensations or improving cognitive abilities—but also have a positive impact on psychosocial aspects. This is particularly important for older adults, whose quality of life is often impaired by physical limitations or psychological stress. In addition, the literature shows a clear link between motor changes in old age and the increased risk of road traffic accidents. Crossing roads safely requires not only physical skills such as speed and coordination but also cognitive processes such as decision-making and risk assessment. It is therefore crucial that future research continues to focus on the development of effective VR training programs to specifically promote these skills. It remains uncertain to what extent VR training actually leads to improved performance in crossing streets or reduces the risk of accidents. Further research is necessary to confirm these significant findings in a representative manner. This relates closely to the concept of fidelity in VR, which influences transfer of training. High perceptual and functional fidelity increase the likelihood of behavioral transfer to real-world contexts [43,44].

Overall, it appears that virtual reality is a promising tool for supporting older adults in their mobility and increasing their safety on the road. Through targeted training programs in virtual environments, seniors can not only improve their skills but also strengthen their self-confidence and thus increase their quality of life.

In summary, it can be said that virtual reality is a promising tool for improving the safety of older people in road traffic. Innovative training approaches can be used not only to train motor skills but also to promote self-confidence and reduce the fear of mobility restrictions in old age.

### Limitations

Several limitations should be noted. First, the study did not include a direct comparison between VR performance and real-world street-crossing behavior, nor did it conduct outdoor or in situ assessments to validate the VR findings. While the VR system logged behavioral metrics such as head rotation and walking speed, these data were not systematically analyzed or compared to real-world benchmarks. Future research should incorporate a control group and/or randomized controlled design, as well as systematic validation of VR outcomes against real-world observations, to further establish the ecological validity of the intervention and strengthen the interpretation of behavioral metrics. No long-term follow-up was conducted to assess whether improvements were sustained over time, which should be addressed in future trials.

Moreover, the participant sample lacked diversity in terms of socioeconomic status and cultural background, which may limit the generalizability of findings.

Due to the limited final sample size (*n* = 20), findings must be interpreted with caution. While results show promising trends, larger and controlled trials are necessary to confirm these effects and rule out sampling bias. Given the exploratory nature of this pilot study, *p*-values are reported descriptively and should not be interpreted as confirmatory without replication in larger trials.

Future research should involve randomized controlled trials with larger samples to validate the observed effects and establish causal inferences.

## 5. Conclusions

The increasing integration of virtual reality (VR) technologies into healthcare opens up numerous innovative approaches to improving the quality of life of older adults. These technologies offer opportunities not only for cognitive stimulation but also for promoting emotional well-being and social interaction. The VR application “Wegfest” exemplifies how such technologies can be used in a targeted manner to address specific challenges in the everyday lives of senior citizens.

Studies show that VR applications can have significant positive effects on cognitive function and balance in older people. A study by Appel et al. (2020) [45] found that immersive VR experiences can improve the subjective well-being and cognitive performance of seniors with mild cognitive impairment. These findings highlight the potential of VR as a therapeutic tool that not only promotes cognitive stimulation but also enhances emotional well-being by enabling users to activate positive memories and promote social interactions [46,47]. In addition, a study by Bruun-Pedersen et al. (2016) [48] has shown that VR technologies can motivate residents of care facilities to exercise more. This is particularly important as physical activity has been shown to reduce the risk of falls and improve the general health of older people. The combination of exercise and VR can therefore be an effective strategy to promote both physical and mental health.

Another key aspect of the use of VR by senior citizens is the improvement of social interaction. According to Zhou et al. (2023) [49], Tabbaa et al. (2019) [50], VR applications can help older people communicate about their experiences and actively participate in social activities. This is particularly relevant in times of social isolation, as experienced during the COVID-19 pandemic. The ability to take virtual trips or participate in interactive group activities can help reduce loneliness and increase a sense of belonging [31].

Despite the promising benefits, there are also challenges and ethical considerations associated with the use of VR technologies in older people. It is important to ensure that these technologies are not only effective but also used safely and ethically [49] emphasized the need for responsible use of data and protection of user privacy. In addition, potential risks such as nausea or disorientation should be considered when using VR [35].

## 6. Outlook

Virtual reality technologies offer promising potential to support health behavior and improve quality of life among older adults. The “Wegfest” application illustrates how VR can be used to address specific challenges such as safe road crossing. Future research should examine how VR training parameters influence real-world behavior and how evidence-based care models can incorporate such tools.

An interdisciplinary approach—combining expertise from healthcare, psychology, and computer science—is essential for developing effective and user-friendly applications. Understanding user interaction, overcoming technological barriers, and offering training programs can enhance acceptance and accessibility. To support the integration of VR technology into healthcare, we recommend interdisciplinary research frameworks such as Design-Based Research (DBR) and the Participatory Action Research (PAR). These methodologies promote close collaboration between technical and healthcare experts, iterative co-design with end users, and rigorous evaluation of both usability and clinical outcomes.

In summary, VR has strong potential to promote mobility, independence, and well-being in older adults. Future studies should further evaluate both the effectiveness and integration of such applications into routine care structures.

## Figures and Tables

**Table 1:** Representation of the parameters and respective gradations in path fixed.

Parameters	Difficulty Level			
	1	2	3	4
Lane width	Single-track	Two-lane	Single lane with cycle path	Two-lane crosswalk with cycle path
Crossing aids	Traffic light	crosswalk	Traffic island	No crossing aid
Traffic volume	low	medium	high	-
Speed zones	30 km/h zone	50 km/h zone	70 km/h zone	-
Times of day	day	twilight	Night	-
Electromobility	low	medium	high	-

Note: The total scene difficulty score is calculated as the sum of the assigned values for each parameter.

**Table 2:** Configuration of a scene in “Wegfest”.

Scene	Lane Width	Speed Zones	Crossing Aids	Time of Day	Traffic Volume	Electromobility	Score
1	1	1	1	1	1	1	6
2	1	1	1	1	1	2	7
3	1	1	1	1	2	1	7
4	1	1	1	2	1	1	7
5	1	1	2	1	1	1	7
6	1	2	1	1	1	1	7
7	2	1	1	1	1	1	7
							48

**Table 3:** Pre- and post-intervention results for main outcome measures (*n* = 20).

Outcome	Pre (Mean ± SD)	Post (Mean ± SD)	*p*-Value	Cohen’s d
TUG (s)	12.1 ± 2.2	10.6 ± 2.0	0.002	0.78
FES-I (score)	27.8 ± 4.1	24.9 ± 3.8	0.005	0.65
MoCA (score)	25.3 ± 2.7	25.5 ± 2.8	0.56	0.10
Subjective safety (VAS)	4.2 ± 1.1	6.8 ± 1.2	<0.001	2.18

Legend: TUG = Timed Up and Go; FES-I = Falls Efficacy Scale-International; MoCA = Montreal Cognitive Assessment; VAS = Visual Analogue Scale (0–10).

## Data Availability

Original data are available from the authors upon request.

## Abbreviations

The following abbreviations are used in this manuscript:

AR	Augmented Reality
FES-I	Falls Efficacy Scale-International
MCI	Mild Cognitive Impairment
MoCA	Montreal Cognitive Assessment
MR	Mixed Reality
RCT	Randomized Controlled Trial
TUG	Timed up and Go test
VAS	Visual analogue scale
VR	Virtual Reality

## Appendix A

**Table A1:** Comparative overview of VR-based training applications for older adults.

Study	Locomotion	Interaction	Adaptive Logic	Study Design	Results/Outcomes
Optale et al. (2010) [8]	Seated	Joystick navigation, voice input	Progressive levels	RCT, *n* = 36	Improved memory and global cognitive functioning in MCI patients.
Anderson-Hanley et al. (2012) [6]	Stationary cycling	Pedal movement, handlebars	Fixed difficulty	RCT, *n* = 102	Improved balance and coordination.
Cho et al. (2014) [9]	Standing	Wii Fit balance board	Fixed difficulty	RCT, *n* = 32	Enhanced balance performance measured by Romberg test; significant improvements in postural control.
Mirelman et al. (2016) [10]	Treadmill walking	Projected visual stimuli	Adaptive difficulty	RCT, *n* = 302	42% reduction in fall risk over 6 months in older adults using treadmill with VR training compared to treadmill alone.
Liao et al. (2019) [11]	Seated/standing	Headset, motion sensors	Scenario-based	RCT, *n* = 34	Significant improvements in global cognition, delayed verbal memory, and instrumental activities of daily living (IADL) in older adults with mild cognitive impairment (MCI).
Paletta et al. (2019) [12]	Walking in place/seated	Headset gaze, simple input	No adaptive logic	Pilot, *n* = 12	Positive emotions, improved quality of life, social interaction.
Szczepocka et al. (2024) [13]	Seated	Headset, controller	Progressive tasks	RCT, *n* = 72	Positive changes in cognitive functioning, including attention and visual memory, in healthy older adults after VR-based cognitive training.
Kustanovich et al. (2023) [5]	Seated/standing	Controller-based tasks	Predefined task progression	10-week intervention	Improved memory and cognitive function in dementia patients

Note: Abbreviations—RCT = Randomized Controlled Trial; MCI = Mild Cognitive Impairment. Studies are listed in chronological order.

Legend:

- Locomotion: How users move within the virtual environment (e.g., physical walking, cycling, standing).
- Interaction: Methods for interacting with the VR system (e.g., controllers, hand tracking, gaze).
- Adaptive Logic: Whether and how the application adapts to user performance or needs.
- Study Design: Type and size of study.
- Results/Outcomes: Key findings relevant to mobility, cognition, or well-being.
